# MerTK is a novel therapeutic target in gastric cancer

**DOI:** 10.18632/oncotarget.3750

**Published:** 2015-04-20

**Authors:** Jun Ho Yi, Jiryeon Jang, Jeonghee Cho, In-Gu Do, Mineui Hong, Seung Tae Kim, Kyoung-Mee Kim, Sujin Lee, Se Hoon Park, Joon Oh Park, Young Suk Park, Won Ki Kang, Ho Yeong Lim, Jeeyun Lee

**Affiliations:** ^1^ Division of Hematology-Oncology, Department of Medicine, Samsung Medical Center, Sungkyunkwan University School of Medicine, Seoul, Korea; ^2^ Samsung Genome Institute, Seoul, Korea; ^3^ Department of Pathology, Kangbuk Samsung Medical Center, Sungkyunkwan University School of Medicine, Seoul, Korea; ^4^ Department of Pathology and Translational Genomics, Samsung Medical Center, Sungkyunkwan University School of Medicine, Seoul, Korea; ^5^ Division of Hematology-Oncology, Department of Medicine, Hanyang University Hospital, Seoul, Korea

**Keywords:** gastric cancer, MerTK, patient-derived cells

## Abstract

**Introduction:**

The role of MerTK has not been assessed in gastric cancer (GC). The aim of this study was to identify a subgroup of GC patients with MerTK tumor overexpression, and to evaluate MerTK as a potential therapeutic target in this disease.

**Methods:**

Protein and mRNA expression of MerTK were evaluated, and other various *in vitro* analyses including shRNA transfection, cell cycle anslysis, MTS assay and colony forming assay were carried out with GC cell lines and GC patient-derived cells (PDCs).

**Results:**

shRNA-mediated knockdown of MerTK resulted in inhibition of cell growth, as well as increased cellular apoptosis in MerTK positive GC cells. Out of 192 GC patients, 16 (8.3%) patients showed strong protein expression and they had a significantly shorter overall survival compared to those with no MerTK expression. In 54 cases of GC PDCs, 4 cases (7.4%) showed mRNA overexpression, which was comparable to the protein expression rate. When we administered UNC1062, a novel MerTK-selective small molecular tyrosine kinase inhibitor, proliferation of MerTK overexpressing GC cells and PDCs were considerably inhibited.

**Conclusion:**

MerTK may be involved in GC carcinogenesis, and it could be a potential novel therapeutic target in GC patients.

## INTRODUCTION

Gastric cancer (GC) is one of the most lethal malignancies worldwide, with an incidence of 18.9/100, 000 cases per year and a mortality rate of 14.7/100, 000 per year [1]. While complete surgical resection is the primary treatment for potential cure, approximately 50% of the patients eventually have unresectable or metastatic disease [2, 3]. Although chemotherapy significantly improved survival in comparison to best supportive care in these patients [4], the benefits of cytotoxic agents are limited to just over 1 year [5, 6]. After molecular targeted agents have been introduced for treatment of GC, trastuzumab which targets HER-2 (human epidermal receptor-2), and ramucirumab which targets VEGFR-2 (vascular endothelial growth factor receptor-2) proved their clinical efficacies in large clinical trials [7–9]. Despite of these successes, the pathogenesis of GC is poorly understood, and there are still unmet needs for the novel therapy.

A number of receptor tyrosine kinases (RTKs) are implicated in the pathogenesis of cancer, and among them, aberrant activation of the TAM (Tyro-3, Axl, and MerTK) family of RTKs is known to be associated with pathogenesis of several malignancies, including melanoma [10, 11], leukemia [12–14], and glioma [15, 16]. They share structural similarity with a combination of 2 immunoglobin-like domains and dual fibronectin type III repeats in the extracellular region and a cytoplasmic kinase domain, and are involved in innate immune response [17], angiogenesis [18] and regulating nervous systems [19]. While several point mutations have been reported, most cases of abnormal receptor activation identified in tumors probably resulted from abnormal expression of either TAM kinases or their ligands, growth arrest specific gene 6 (Gas6) [20]. Although the detailed molecular mechanism underlying TAM RTKs-driven carcinogenesis remains unclear, previous *in vivo* and *in vitro* studies have suggested that activated anti-apoptotic pathways mediated by PI3K and MAPK via the TAM RTKs may play a crucial role in this mechanism.

Whilst several studies have suggested potential carcinogenic role of Axl overexpression in solid tumors [21–23], the role of MerTK has yet been understood, especially patients with GC. In the current study, we have found that shRNA-mediated knockdown in MerTK(+) GC cells led to a considerable growth inhibition and apoptosis. In addition, we reviewed surgical specimens of 192 GC patients and found that a subset of these patients has MerTK protein overexpression, and that this in turn is significantly association with a poor clinical outcome. Furthermore, we examined the efficacy of UNC1062, a novel MerTK-specific small molecule, in MerTK (+) GC cell lines and patient derived cells (PDCs) from MerTK overexpressing GC. Taken together, our data suggest that MerTK may be involved in carcinogenesis of GC, and could be a potential target for drug development in a subset of GC patients.

## RESULTS

### GC cell line screening for MerTK overexpression

**Figure 1:**
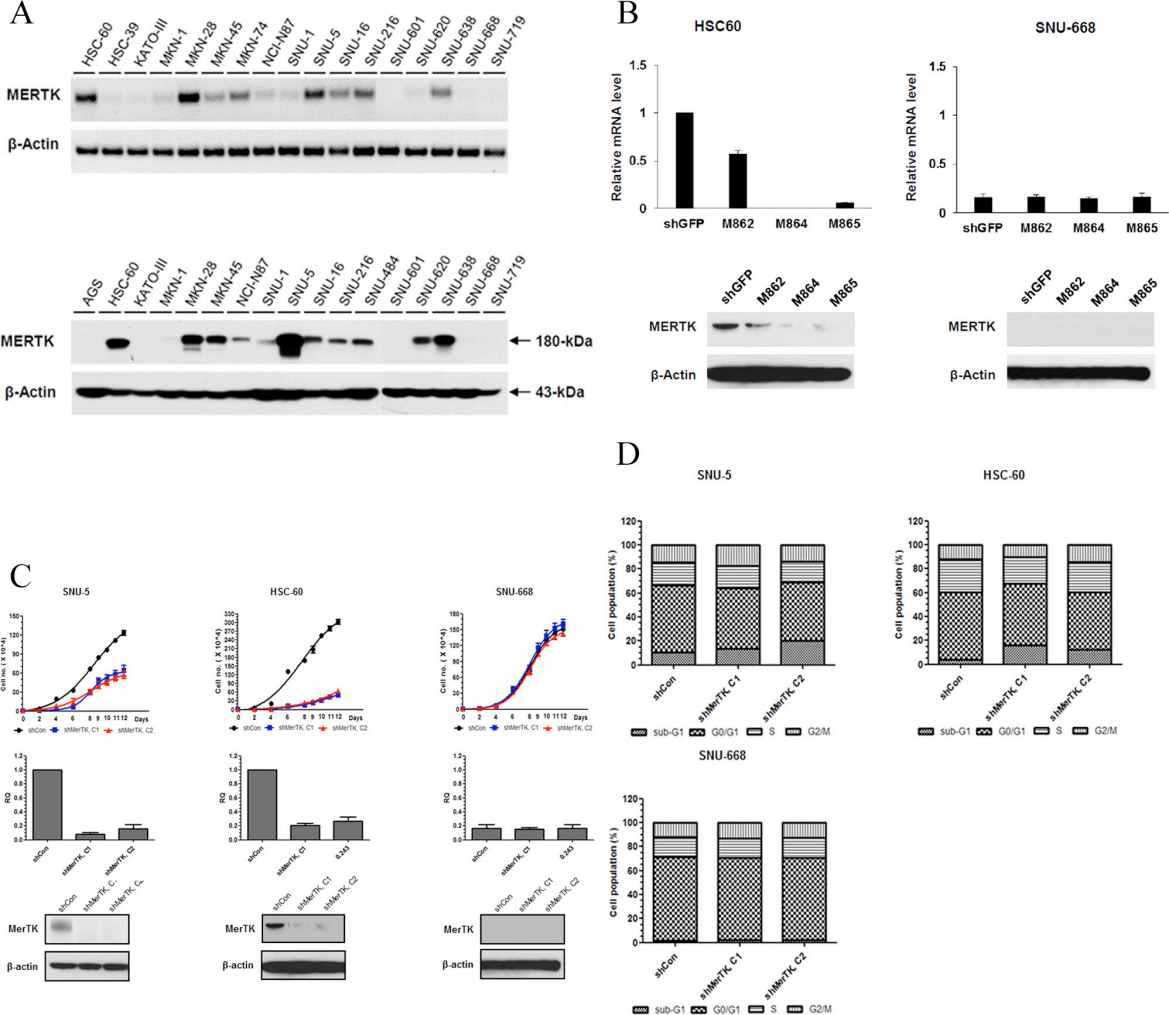
A. Screening for MerTK positive GC cell lines (Upper, RT PCR; lower, western blot) **B.** In HSC-60 (MerTK-overexpressing) cells, shRNA (M864, M865)-mediated knockdown resulted in decreased expression of MerTK mRNA and protein. In SNU-668 cells (MerTK negative), neither the level of mRNA nor protein changed after lentiviral infection. **C.** The proliferation of two MerTK-positive cell lines (SNU-5 and HSC-60) was significantly inhibited by MerTK targeting shRNA, whereas proliferation of SNU-688 was not affected **D.** Transfection with MerTK-specific shRNA significantly increased the apoptotic fraction of SNU-5 and HSC-60 cells, compared to a control (M862) clone (16.3%, 12.5%, and 4.1% in cells transfected with M864, M865, and M862, respectively). The apoptotic fraction of SNU-668 cells was unchanged.

To investigate the functional role of overexpressed MerTK in GC, we sought cell lines expressing high levels of MerTK by screening 17 GC cell lines by using RT-PCR and western blot (Figure 1A). Based on these data, we selected HSC-60 and SNU-5 as MerTK-positive cell lines, and SNU-668 cells as a MerTK-negative cell line, for further experiments.

### Knockdown of MerTK causes cell death via inducing apoptosis

Next, by lentiviral infection, we introduced three vectors encoding shRNAs (M862, M864, and M865) targeting the sequences of *MerTK* into HSC-60 and SNU-668 cells along with a control shRNA targeting *GFP*. Compared to the control shRNA, we found that 2 shRNAs (shM864 and M865) reduced the expression levels of *MerTK* mRNA as well as MerTK protein in HSC-60 cells by more than 80% as determined by quantitative RT-PCR and western blot analysis, respectively, confirming the specific ablation of MerTK (Figure 1B). The growth of HSC-60 cells was significantly inhibited by both the M864 and M865 clones compared to that of control shRNA-treated colony (*P* < 0.001). A similar effect on growth was observed for another MerTK-positive cell line, SNU-5. As a control, we transfected the non-MerTK expressing SNU-668 cells with the M864 and M865 clones, but this had no effect on cell growth (Figure 1C).

We next examined whether knockdown of MerTK affects the cell cycle. Transfection with M864 or M865 significantly increased the sub-G1 fraction, that is, the fraction of apoptotic cells, compared to the control (M862) clone. (16.3%, 12.5%, and 4.1% in cells transfected with M864, M865, and M862, respectively; Figure 1D). In contrast, there was no significant increase in the sub-G1 fraction of M862-, M864-, or M865-transfected SNU-668 cells. Collectively, these results suggest that MerTK plays an important role in cell growth as well as cell cycle progression in a subset of GC cell lines that overexpresses MerTK.

### MerTK protein overexpression predicted poor prognosis in GC

**Figure 2:**
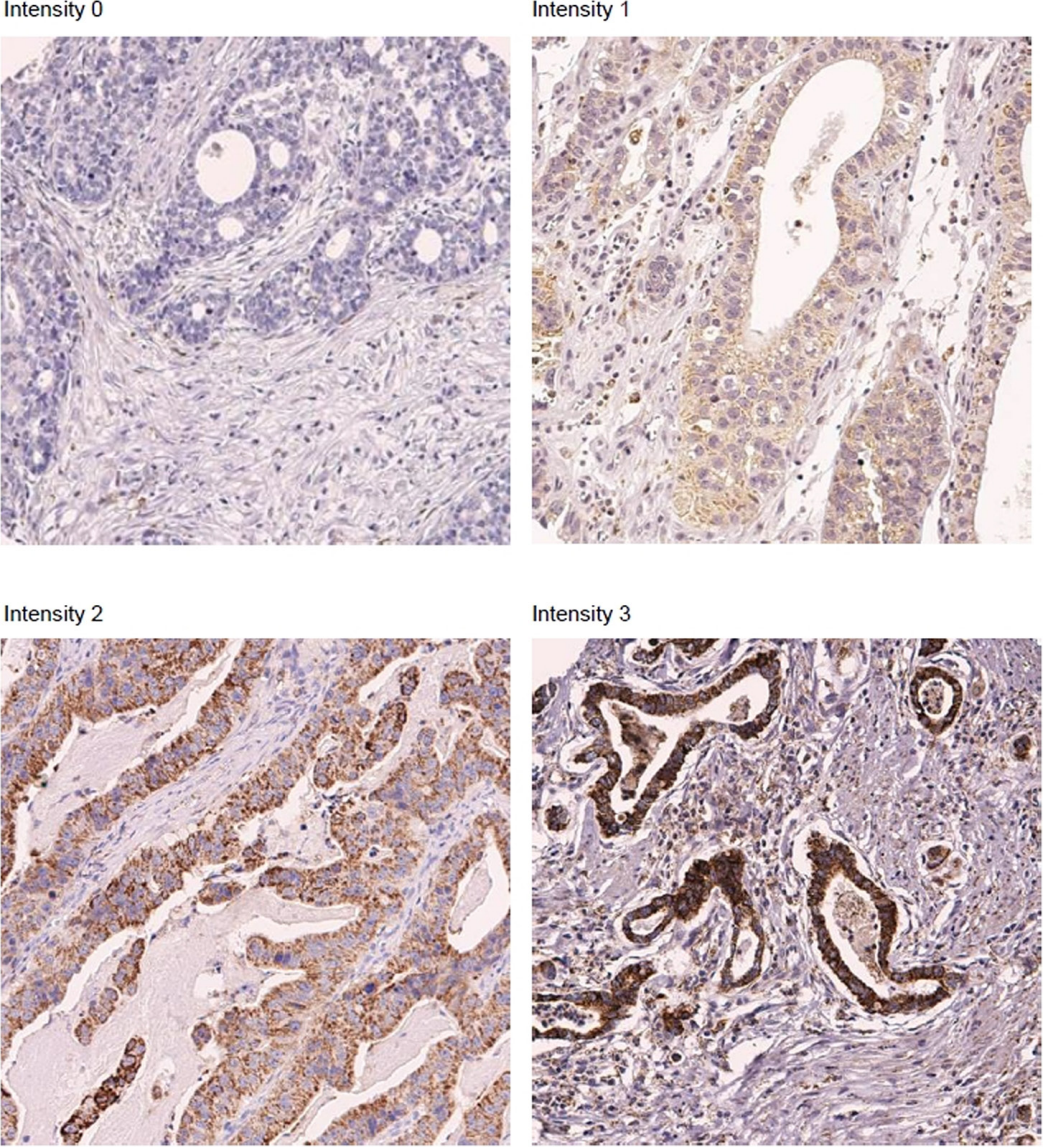
Representative sections showing MerTK overexpression on immunohistochemical studies. (× 200)

To examine the role of MerTK in patients with GC, we performed a retrospective analysis of 192 GC patients. The baseline characteristics are described in Table 1. MerTK overexpression (characterized as IHC intensity 2–3) was present in 16 patients (8.3%), and weak expression (intensity 1) was identified in 11 patients (5.7%). Representative sections showing MerTK overexpression are shown in Figure 2. Regarding the mRNA expression, 4 of 54 cases (7.4%) of GC-derived PDCs demonstrated MerTK overexpression by the NanoString-based multigene assay. Notably, of the 4 cases for which MerTK mRNA overexpression was identified, all of the primary tumors were characterized by IHC to have strong MerTK overexpression (*data not shown*).

**TABLE 1:** Baseline characteristics of the MerTK-IHC cohort (*n* = 192)

	*N* (%)
Age, median (range)	51 (28 – 74)
Sex	
Male	120 (62.5)
Female	72 (37.5)
Type of gastrectomy	
Subtotal gastrectomy	131 (68.2)
Total gastrectomy	61 (31.8)
WHO Histologic classification	
Well to moderately differentiated	51 (26.6)
Poorly differentiated	79 (41.1)
Signet ring cell	56 (29.2)
Mucinous	5 (2.6)
Others	1 (0.5)
Lauren’s classification	
Intestinal-type	62 (32.3)
Diffuse-type	123 (64.1)
Mixed-type/Unknown	7 (3.6)
Lymphovascular invasion	
Present/identified	93 (48.4)
Not present/Not identified	99 (51.6)
Pathologic stage^*^	
IB	32 (16.7)
II	76 (39.6)
IIIA	50 (26.0)
IIIB	8 (4.2)
IV	26 (13.5)

^*^According to AJCC 6th edition

**Figure 3:**
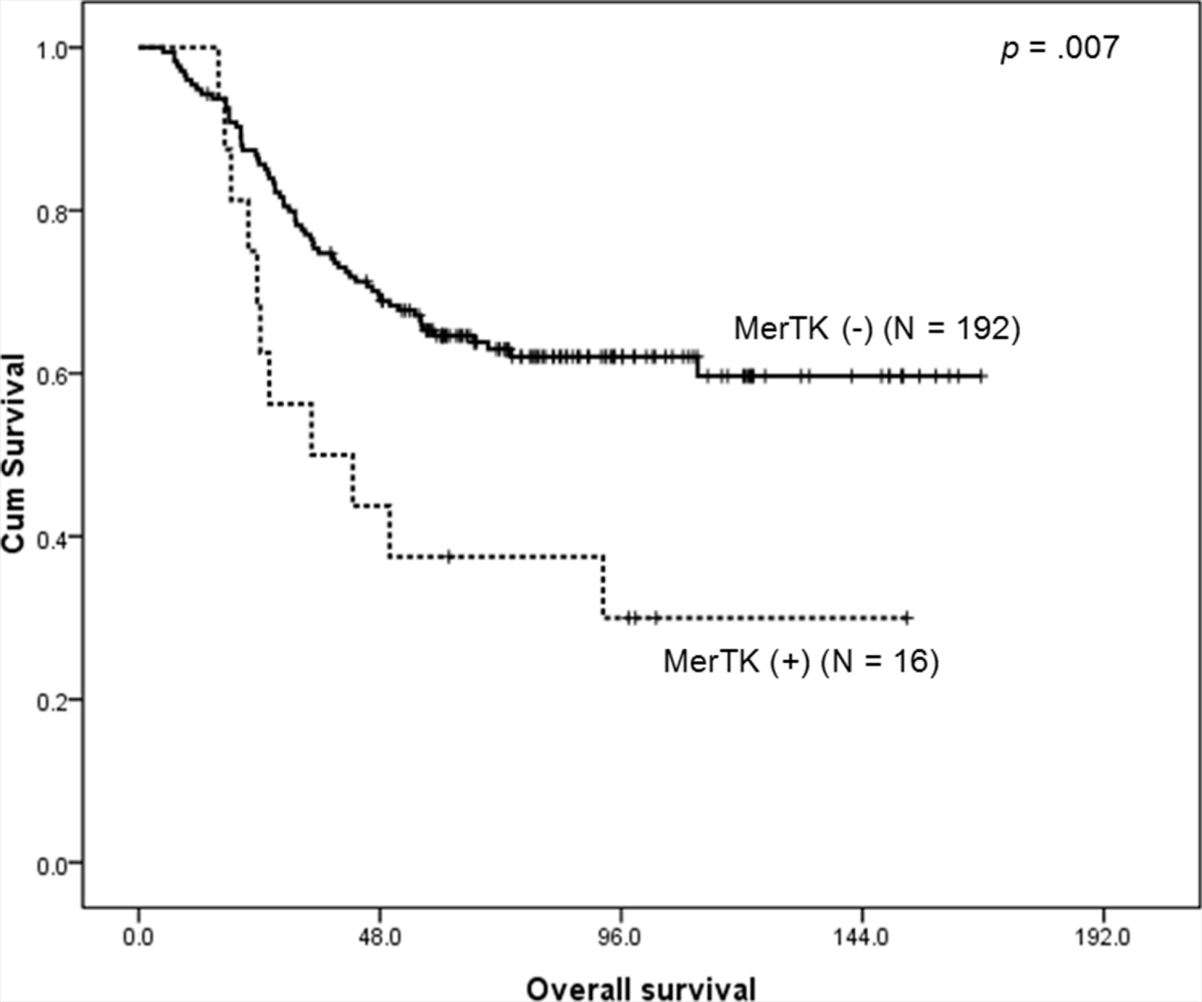
Kaplan-Meier survival curve for overall survival according to the MerTK status demonstrates that patients with MerTK overexpressing tumors have a worse outcome

With the median follow-up duration of 107.6 months (95% confidence interval (CI) 100.5 – 114.7 months), MerTK overexpressed GCs were associated with a significantly shorter OS (median OS, 34.4 months, 95% CI, 8.6 – 66.9 months) when compared to those without MerTK overexpression (median OS not reached, *p* = 0.007) (Figure 3). For further characterization of MerTK-positive GC patients, we performed subset analysis. The patients with MerTK overexpression were more likely to have intestinal-type tumors (81.3% *vs*. 27.8%, *p* < 0.0001), and advanced stage tumors (68.8% *vs*. 41.5%, *p* = 0.036; Table 2). Multivariate analysis revealed that MerTK overexpression was associated with poor OS (hazard ratio [HR] 5.50, 95% CI 2.41 – 12.59, *p* < 0.001), along with advanced-stage tumors, (HR 2.79, 95% CI 1.61 – 4.85, *p* < 0.001), and diffuse-type tumors (HR 2.29, 95% CI 1.16 – 4.50, *p* = 0.017; Table 2).

**TABLE 2:** Clinicopathological characteristics of MerTK(+) GC patients and the multivariate analysis for overall survival

Clinicopathologic features of MerTK(+) GC patients			
	MerTK positive (*N* = 16)	MerTK negative (*N* = 176)	*p*
Age, < = 60	11 (68.8%)	127 (72.2%)	0.716
**Male**	**14 (87.5%)**	**106 (60.2%)**	**0.059**
Lauren’s classification			
Intestinal-type	13 (81.3%)	49 (27.8%)	< 0.0001
Diffuse-type	3 (18.8%)	120 (68.2%)	
Others	0 (0.0)	7 (4.0%)	
Stage			
Stage Ib/II	5 (31.3%)	103 (58.5%)	0.036
Stage III/IV	11 (68.8%)	73 (41.5%)	
Lymphovascular invasion (+)	7 (43.8%)	85 (48.3%)	0.072
**Multivariate analysis for overall survival**			
	**HR**	**95% CI**	***p***
**Advanced stage (III/IV)**	**2.79**	**1.61 – 4.85**	**< 0.001**
**Diffuse-type cancer**	**2.29**	**1.16 – 4.50**	**0.017**
**MerTK overexpression**	**5.50**	**2.41 – 12.59**	**< 0.001**
**Male**	**1.025**	**0.57 – 1.83**	**0.934**
**Lymphovascular invasion**	**1.48**	**0.84 – 2.61**	**0.176**

### Effects of UNC1062 on GC cells

**Figure 4:**
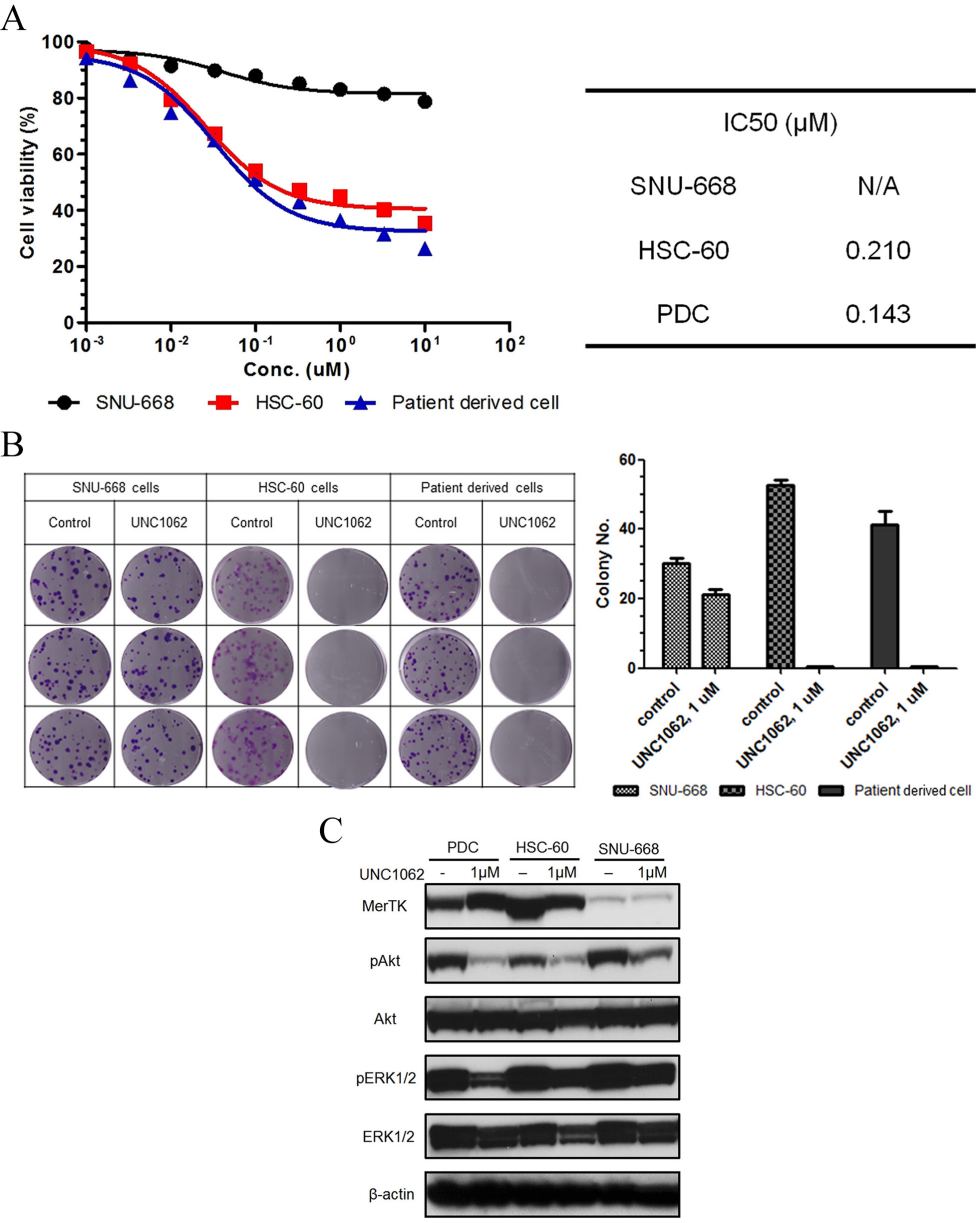
A. Results of the MTS assay for a MerTK-specific inhibitor (UNC1062). Both HSC-60 and MerTK-positive patient-derived cells (PDCs) had similar sensitivities and IC50 values The cellular viability of SNU-668 was not affected by UNC1062. B. The colony formation of HSC-60 and MerTK-positive PDCs was completely abolished by UNC1062, but SNU-668 was unaffected. C. When cells were treated with UNC1062, phosphorylation of AKT and ERK1/2 were decreased in HSC-60 and MerTK positive PDCs, suggesting they are downstream output pathways of MerTK.

To evaluate effects of UNC1062, a novel MerTK-specific small molecular TKI, on GC cells, we performed a cell viability assay using commercial GC cell lines and PDCs with MerTK mRNA overexpression, as identified by the nCounter assay. The MerTK-positive PDCs showed similar sensitivity to UNC1062 and IC50 values similar to those of HSC-60 (Figure 4A). And to characterize the functional consequences of MERTK-mediated pro-survival signaling, we carried out colony forming assay (Figure 4B). The vehicle untreated SNU-668, HSC-60, and PDCs formed 30.00 ± 0.333, 53.00 ± 0.333, and 41.00 ± 0.8819 colonies, respectively. When UNC1062 was administered, SNU-668 cells formed an average of 21.00 ± 0.333 colonies while colony formation of both HSC-60 and the PDCs was abolished. By western blot, we observed that inhibition of the MerTK pathway by UNC1062 resulted in the inhibition of downstream phosphorylation of AKT and ERK1/2 (Figure 4C). Again, we observed a similar pattern between PDCs and HSC-60.

## DISCUSSION

In the current study, we have found that 1) MerTK-specific shRNA knockdown resulted in considerable growth inhibition and inducing apoptosis in MerTK-overexpressing GC cell lines; 2) MerTK protein overexpression by IHC was found in 8.3% (16/192) of GC patients, who were associated with a poor survival duration; 3) MerTK mRNA overexpression was found in 7.4% (4/54) cases of GC PDCs, which was comparable with the MerTK protein overexpression; and 4) UNC1062 inhibited tumor growth of MerTK-overexpressing cell lines and PDCs. Importantly, these effects specifically occurred in a cell line expressing high levels of MerTK, suggesting that targeting MerTK could be a novel therapeutic option in MerTK overexpressing GC.

Despite of recent advances of early screening methods and treatments, GC still remains as one of the deadliest disease. Even after extended surgical resection followed by proper adjuvant treatment, over 30% of patients will experience disease recurrence or cancer-related death [3, 25]. Regarding the genomic profiles of GC patients, two large-scaled whole-exome sequencing studies have shown that only two genes are found as commonly mutated between two studies [26, 27], pointing the heterogeneity of the disease. And using various genomic analytic tools, a study by Deng et al. has found that at least 37% of GC patients may be treated by RTK directed therapies [28]. These findings necessitate precise approaches in treatment of GC patients.

After first identified in leukemic lymphocytes [29], the function of MerTK has been steadily investigated. While several somatic mutations of MerTK were found in certain cancers [30], it is believed that their functional role is limited, and ectopic expression or overexpression seems to be the major pathogenesis in various neoplasms [10, 12–14, 31]. Gas6, the ligand of TAM RTK family, is also known to act as a tumor mitogen [32]. In several animal studies, it was suggested that TAM RTKs could be an oncogenic driver [33, 34]. On the other hands, several recent studies have shown that TAM RTKs rather act as a manner of “non-oncogene addiction”, that is, providing favorable stimuli on tumor survival under stressful conditions which causes chemoresistance [12, 15, 31] or promoting tumor metastasis by suppressing natural killer (NK) cell function [35, 36]. Our findings that the growth of MerTK positive GC cells was profoundly inhibited by either MerTK-specific shRNAs or a small molecular inhibitor suggest that MerTK positive GC cells might depend on MerTK for their survival. It is also consistent with previous studies that MerTK specific inhibition resulted in increased incidence of cellular apoptosis.

Unlike other solid tumors, the prognostic role of MerTK has not been extensively studied in GC. Its expression was first identified in several GC cell lines in 1998 [37]. After that, only one published report has shown that the co-expression of MerTK and Axl was associated with adverse outcomes in a series of 96 GC patients [38]. Our experimental results are consistent with this finding. Among our IHC validation cohort consisting of 192 GC patients treated with curative surgery followed by adjuvant chemoradiation, 16 patients (8.3%) had tumors with increased MerTK signal by IHC, and they had significantly short OS. And this result is comparable to those of the MerTK mRNA overexpressing population identified by the Nanostring assay (4 of 54, 7.4%). Moreover, those who had MerTK overexpressed GC demonstrated unique clinicopathologic features, that is, they were more frequently associated with an intestinal-type cancer and an advanced stage. Multivariate analysis revealed that MerTK protein expression was associated with poor OS, along with other known prognostic factors, including an advanced stage and a diffuse-type cancer, by Lauren’s classification.

In terms of MerTK immunohistochemical analysis in GC patients, there are no validated criteria. In Wu et al.′s report [38], if positive stained cells are more than 25% of all tumor cells, it was interpreted as positive protein expression. With this criterion, 60 out of 96 cases (62.5%) were regarded as MerTK expressing tumors, which looks somewhat high in rate. In the current study, however, we used both intensity scale and proportion scale. The criteria for positive stain were 2 or 3 in intensity and positive stains in more than 2 out of 4 tissue cores. Moreover, as we validated the criteria through clinicopathologic characterization and survival analysis, this could be used in the future analysis for MerTK in GC patients.

Diverse downstream signals of MerTK have been identified, including MAPK/ERK, AKT, and PLCγ pathways [39–42] which differ according to cell type and tissue microenvironment [43]. In gastric cancer, there is one report that administration of recombinant Gas6 phosphorylated Axl in GC cell lines, and then enhanced cellular survival and suppressed apoptosis via Akt pathway [44]. In agreement with this report, we found that both phospho-ERK and phospho-AKT were significantly reduced by MerTK inhibition, suggesting that they are downstream signaling pathways in gastric cancer cells.

Owing to the recent discovery of the significance of TAM RTKs, several TAM-specific inhibitors are currently in development [10, 45–48], and in the current analysis, we used UNC1062. UNC1062 is a potent and selective MerTK inhibitor with minimal effects on potassium channel, which could cause long QT syndrome [24]. In a study dealing with the MerTK (+) melanoma cells [11], UNC1062 induced cell death similar to the current study, and it also inhibited migration and invasion of the melanoma cells. In pharmacologic point of views, this agent has demonstrated a potent anti-MerTK effect, particularly in a human leukemia cell line (IC50 of 1.1 nM), and a somewhat lower effect in human solid cancer cell lines (around 300 nM) [24]. The IC50 values from our analyses on GC cell lines and PDCs were 0.21 μM and 0.14 μM, respectively. Although the levels are slightly higher than that of the leukemia cell line, treatment with 1.0 μM UNC1062 was sufficient to completely abrogate colony formation of GC cell lines and PDCs. While several approved agents including bosutinib, crizotinib and vandetinib also contain anti-TAM RTK efficacies [46], the effects of TAM-directed therapy have yet been evaluated in the clinical setting. Further analysis of their therapeutic roles is required.

The present study has several limitations. Regarding the retrospective analysis, patients with metastatic disease were not included. If MerTK is associated with tumor survival and metastasis, patients with metastatic disease may show more frequent MerTK expression. And as with study by Wu et al. [38], only Asian patients were included which warrants further analysis in the Western population. And as MerTK is known to be involved chemoresistance, additional analyses regarding the combined treatment are required.

To our knowledge, the current study provided with useful information to define prognostic and functional roles of MerTK in GC for the first time. The unique features of MerTK (+) GC patients could provide with further characterization of GC population, and it might be a potential therapeutic target as well. The analysis for role of MerTK inhibitors, including UNC1062, in GC patients should be continued including prospective clinical trials.

## MATERIALS AND METHODS

### Establishing PDCs, cell culture, and reagents

To establish PDCs from metastatic GC patients with malignant effusion, those who were enrolled onto the SMC Oncology Biomarker study (NCT#01831609) were screened for the MerTK overexpression by Western blot. All patients provided informed consent form according to the SMC Institutional Review Board. Briefly, collected effusions (1–5 L) were divided into 50 mL tubes, centrifuged at 1500 rpm for 10 min, and washed twice with PBS. Either PDCs or commercial GC cell lines, which were purchased from the Korea Cell Line Bank (KCLB, Seoul, Korea), all cells were grown in RPMI-1640 medium (PAA Laboratories GmbH, Austria) supplemented with 10% heat-inactivated fetal bovine serum, an antibiotic, and an antimycotic. Cells were incubated at 37°C in 5% CO_2_ and the medium changed twice a week. After becoming confluent, cells were subdivided into new flasks until the end of the experiment.

### Western blot analysis

Total cell extracts were obtained using a lysis buffer (20 mM HEPES pH 7.4, 150 mM NaCl, 1 mM MgCl_2_, 1 mM EDTA, 2 mM EGTA, 10% glycerol, 1% Triton X-100, 1 μg/mL leupeptin, and 1 μg/mL aprotinin). Equal amounts (40 μg) of cell lysates were resolved on 8% or 12% Bis-Tris gels with MOPS running buffer (Invitrogen, Novex), transferred to PVDF membranes, and incubated with specific antibodies against MerTK (Abcamplc, Cambridge, UK) and beta-Actin (Santa Cruz Biotechnology, Santa Cruz, CA, USA). Immune complexes were visualized as enhanced chemiluminescence (Novex ECL, Invitrogen).

### RT-PCR

cDNA were generated by reverse-transcription of purified RNA, using the Omniscript RT kit (Qiagen, Hilden, Germany) following the manufacturer’s protocol (60 min reaction at 37°C). The MerTK forward primer sequence was 5′-CGAGCTCGGATCTCTGTTCA-3′, and the reverse primer sequence was 5′-GAGGGGGCATAATCTACCCA-3′, which resulted in a 269-bp amplicon. A 300-bp amplicon of ACTB was generated as a positive control; the sequence of the forward primer was 5′-TCATCACCATTGGCAAT GAG-3′, and the sequence of the reverse primer was 5′-CACTGTGTTGGCGTACAGGT-3′. The PCR profile was 35 cycles of 95°C for 1 min, 54°C for 1 min, and 72°C for 1 min, with a final extension of 10 min at 72°C.

### Lentivirus production and transduction

MerTK targeting small hairpin RNA (shRNA) and control shGFP were obtained from Sigma Mission shRNA (3 different constructs). VSV-G pseudotyped lentiviruses were produced by co-transfecting 293T cells with the transfer vector and 3 packaging vectors (pMDLg/pRRE, pRSV-REV, and pCMV-VSVG), and the resultant virions were purified by ultracentrifugation. We plated 1 × 10^4^ of HSC-60 or SNU-668 cells in 6-well plates and transduced them with lentivirus, using 8 g/mL polybrene (Sigma).

### Cell growth curve and cycle analysis

We examined the effect of MerTK knockdown on cell growth in HSC-60 and SNU-668 cells. At 72 h post-shRNA transduction, we seeded, in triplicate, samples of cells in log phase growth at a density of 1 × 10^4^ cells/well in 6-well plates. The cells were counted, pelleted by centrifugation at 1300 rpm for 3 min, and re-suspended in fresh media. We counted triplicate sets of cells after 12 d and calculated the mean cell number for each condition. From these data, we derived the growth curves.

Regarding the cell cycle analysis, cultured cells were removed with trypsin and fixed with 70% ethanol at 4°C overnight. Subsequently, they were stained with propidium iodide (20 μg/mL propidium iodide, 200 μg/mL DNase-free RNase A, and 0.1% Triton X-100, prepared freshly in PBS). The cellular DNA complement was analyzed using the FACS Calibur (Becton Dickinson, San Jose, CA). Data were analyzed using the CellQuest software (Becton Dickinson).

### Patients for immunohistochemical study

Among the GC patients who had undergone surgery at Samsung Medical Center from July 1995 to December 2005, 192 were selected based on the following criteria: age ≥ 18 years; pathologically confirmed GC; complete surgical resection of tumor with curative intent; and the availability of formalin-fixed paraffin-embedded tissue suitable for immunohistochemical (IHC) analysis. All patients had undergone gastrectomy with extensive (D2) lymph node dissection, and the treatment was completed by administering 2 more cycles of 5-fluorouracil/leucovorin-based chemoradiation. The pathologic stages were evaluated according to the 6^th^ edition of the staging system published by the American Joint Committee on Cancer (AJCC).

### Tissue microarray construction and IHC stains

Tissue microarrays were constructed using a Beecher Manual Tissue Microarrayer (MTA-1, Beecher Instruments Inc., Wisconsin, USA). All available H&E-stained slides were reviewed, and 4 representative tumor regions were taken from donor formalin-fixed paraffin-embedded blocks using a 0.6-mm core punch, and arrayed into recipient blocks. IHC studies were performed with 4-μm-thick tissue microarray sections by using rabbit anti-MERTK antibody (HPA036196, Sigma Life Science, MO, USA). Tissue microarray sections were deparaffinized 3 times in xylene for a total of 15 min and subsequently rehydrated. Immunostaining was performed using a Bond-max autoimmunostainer (Leica Biosystems, Melbourne, Australia) with Bond™ Polymer refine detection, DS9800 (Vision Biosystems, Melbourne, Australia). Briefly, antigen retrieval was achieved by heating samples to 97°C for 20 min in ER1 buffer, blocking endogenous peroxidase activity with 3% hydrogen peroxidase for 5 min, and incubating samples with a 1:50 dilution of primary antibody for 15 min at room temperature.

Positive signal for MerTK manifested as cytoplasmic or membranous reactivity. The staining intensity was graded on a scale of 0 to 3, where 0 is negative, 1 is weak (only visible at high magnification using a 20× objective), 2 is moderate (readily visible at low magnification, 4× objective), and 3 is strong (strikingly positive, even at low power magnification). When moderate-to-strong reactivity was observed in more than 2 of the 4 cores of tumor cells, it was scored as positive staining. Negative controls (substitution of PBS for the primary antibody) were run simultaneously. The slides were assessed by a pathologist (IG. D.), blinded to the clinical outcome.

### Drug treatment and cellular growth analyses

Cells (3 × 10^3^ in 100µl/well) were seeded on 96-well plates and incubated for 24 h at 37°C and treated with for 3 days UNC1062 at 37°C. After drug treatment, MTS solution was added to each well and incubation was continued for 4 h at 37°C. The absorbance value of each well was measured with a microplate reader set at 490 nm. All experiments were performed in triplicate. UNC1062 inhibitor was kindly provided by Wang et al. [24].

Regarding the colony forming assay, about 2 × 10^2^ cells per well were added to a 6-well culture plate, using three wells for each group. After incubation at 37°C for 14 d, the cells were washed twice with PBS and stained with 0.5% crystal violet in 20% methanol. The number of colonies was counted under a microscope.

### Statistical analysis

The primary end-point for MerTK-IHC analysis was overall survival (OS), defined as the time from surgery to the date of death or the last follow-up, and it was calculated using Kaplan–Meier method. The *χ*^*2*^ test or Fisher exact test was used to determine the strength of associations between MerTK protein expression and different clinicopathological factors. The Cox proportional hazard (backward linear regression) model was used to evaluate the associations between clinicopathological factors and **OS**. All tests were two-tailed, and P values < 0.05 were considered significant. Statistical analysis was performed using SPSS 20 software for Windows (SPSS Inc., Chicago, IL).
